# Tying the Knot for Parents: How Adult Children’s Marital Choices Impact Parental Depression in China

**DOI:** 10.1093/geroni/igaf022

**Published:** 2025-03-08

**Authors:** Xiaowei Dong, Nawi Ng, Lars Jacobsson, Ailiana Santosa

**Affiliations:** School of Aging Services and Management, Nanjing University of Chinese Medicine, Nanjing, China; School of Public Health and Community Medicine, Institution of Medicine, Sahlgrenska Academy, University of Gothenburg, Gothenburg, Sweden; Department of Clinical Sciences, Psychiatry, Umeå University, Umeå, Sweden; School of Public Health and Community Medicine, Institution of Medicine, Sahlgrenska Academy, University of Gothenburg, Gothenburg, Sweden

**Keywords:** Longitudinal study, Mental health, Parent–child, Stress

## Abstract

**Background and Objectives:**

This study aims to examine the causal effect of adult children’s marital status on parental depressive symptoms in the Chinese context, and whether parents’ demographic characteristics play a role in the association.

**Research Design and Methods:**

We utilized the 5 waves of China Health and Retirement Longitudinal Study spanning over 9 years. The participants were categorized into 3 distinct groups: (1) the individuals with at least 1 divorced adult child; (2) those with at least 1 unmarried child aged 30 or older; and(3) a reference group consisting of those not meeting criteria for the first 2 categories. We employed marginal structural models to estimate the causal effect of adult children’s marital status and parental depressive symptoms.

**Results:**

In total, 13,077 participants were included in the analysis. Parents with divorced adult children (β = 1.55, 95% confidence interval [CI]: 1.28–1.82) and those with marriage-delayed children (β = 0.83, 95% CI: 0.53–1.13) had an increased risk of depressive symptoms when compared to the reference group. When the number of divorced or marriage-delayed adult children was included, the coefficient for parental depressive symptoms was 1.10 (95% CI: 0.92–1.28).

**Discussion and Implications:**

This study provides evidence that adult children’s divorce and delayed marriage are associated with parental depressive symptoms in the Chinese context. This research helps uncover culturally significant risk factors for late-life depression, necessitating enhanced psychological support and preventive strategies to address the needs of vulnerable groups.

**Translational Significance:** This study demonstrates a link between adult children’s divorce and delayed marriage with increased depressive symptoms in parents within the Chinese setting. By uncovering culturally relevant risk factors for late-life depression, this research can inform individual interventions and organizational policies aimed at providing targeted psychological support. The findings call for preventive strategies that promote mental health awareness and resilience among aging populations. These efforts could enhance overall well-being and alleviate the mental health burden in later life.

Late-life depression is a significant public health concern due to its harmful effects. It is associated with declines in physical, cognitive, and social functioning, as well as an increased risk of morbidity, suicide, and mortality (Fiske et al., 2009). A meta-analysis reported that the combined prevalence of depression in older adults was 28.4%, despite the limitation of pooling prevalence estimates for both depressive symptoms and major depressive disorder (Hu et al., 2022). The challenges associated with late-life mental health may become even more pronounced as the demographic shift toward an aging population continues to escalate in the future (Van Orden et al., 2015). China is undergoing a rapid aging process and has the world’s largest older population (Chen et al., 2019). Due to the increased dependence of older individuals on their families for support as they age, there has been a notable expansion in the body of literature investigating factors within family dynamics that may contribute to late-onset depressive symptoms (Dong et al., 2023; Sun et al., 2018).

Nevertheless, the reciprocity between family members and older adults is not solely manifested in affection, assistance, and solidarity; it may also act as a stressor for mental health, particularly in the relationship between adult children and parents. In Chinese Confucian philosophy, filial piety, or xiao, refers to the expectation that children should obey and serve their parents. It encompasses a variety of expected behaviors, such as showing respect, ensuring the continuation of the family line, and caring for parents in both sickness and health (Chow, 2001). The term “filial discrepancy” refers to the difference between the behavior of children and the expectations their parents hold for them (Cheng & Chan, 2006). The historical role of older parents in directly arranging or facilitating their children’s marriages has diminished over time. Yet, parental expectations for the attainment of adult roles persist (Chien & Yi, 2012; Jones et al., 2015). Marriage is still perceived as a package of family expectations and obligations for older adults (Raymo et al., 2015). This is exemplified by the increasing popularity of parent-organized matchmaking events in numerous Chinese cities. During these occasions, parents actively promote their adult children’s physical and economic qualities on billboards in public parks, aspiring to establish marriage alliances for their grown children (To, 2013).

When adult children deviate from desirable normative adult statuses aligned with their parent’s expectations, older adults tend to experience psychological distress (Cichy et al., 2013). This distress may stem from intensified feelings of stigma, concern, and fear. Parents may view their children’s deviations as a reflection of their own shortcomings. Mounting evidence suggests that adult children’s divorce may increase the risk of parental depressive symptoms (Fingerman et al., 2012; Kim et al., 2017). However, mixed findings have been reported regarding the impact of adult children’s overage singlehood (Chen & Tong, 2021). Moreover, the existing literature inadequately considers the dynamic nature of confounding and marital status across one to multiple children, limiting the ability to draw causal conclusions. Conventional regression analyses may lead to biased estimates since prior exposure influences the level of the time-dependent confounders (Robins et al., 2000). To address these methodological challenges, the current study adopts marginal structural models (MSMs), which are well suited to account for dynamic exposures and time-dependent confounding. Understanding the impact of multiple children requires flexible models that can distinguish between the effects of a single instance of exposure and cumulative experiences. To explore this, we employed both exposure stress and cumulative stress models to examine how the marital status of multiple adult children affects parental mental health, as well as the potential for incremental effects.

The family life-course perspective centers around interconnected lives, underscoring the interwoven and reciprocally impactful developmental paths shared by parents and adult children that persist throughout their lifespans (Elder et al., 2003). This theoretical framework posits that the life choices and circumstances of grown children may have repercussions on the psychological well-being of their parents (Greenfield & Marks, 2006). Although numerous studies indicated that parental divorce can have adverse effects on the well-being of young children (Sigle-Rushton et al., 2005), there is a growing interest in understanding the reversal influence of adult children’s marital statuses on parents’ mental health (Kalmijn & De Graaf, 2012). Previous empirical research demonstrates that parental gender and being married may influence how stressors affect aging parents (Greenfield & Marks, 2006). We hypothesize that these factors could make certain subgroups of parents less vulnerable to the impacts of their adult children’s divorce or late marriage. Based on the life-course theory, our research aimed (1) to examine the causal effect of adult children’s divorce and delayed marriage on parental depressive symptoms in the Chinese context; and (2) to investigate whether parents’ demographic characteristics (including gender, singlehood, residence area, and education level) play a role in the association.

## Method

### Study Population and Data Source

The China Health and Retirement Longitudinal Study (CHARLS) is a biannual longitudinal survey featuring a nationally representative sample of Chinese adults aged 45 years and older (Zhao et al., 2014). Its primary objective is to delineate the dynamics of retirement and its repercussions on health, health insurance, and economic well-being in China. The data were collected at three levels, namely individual, household, and community levels. The data set includes sociodemographic details, family structure, employment, retirement and pension, income and consumption patterns, individual and household assets, health conditions, functional indicators, health behavior, healthcare, and insurance. The Ethical Review Committee of Peking University approved the data collection process in CHARLS (Zhao et al., 2014). All participants involved in the study provided written informed consent. In this analysis, the sample was limited to individuals aged 50 years or older, all of whom had at least one child aged 18 or older. Respondents without any adult children were excluded from the study.

### Exposure

In this study, each adult child’s marital status was reported by one parent on a household level and we used two stress models to classify the exposures. The exposure model suggests that the presence of even one problematic child can diminish parental well-being. In contrast, the cumulative model posits that an increasing number of issues within the family will exhibit an incremental effect (Fingerman et al., 2012). The exposure stress model was used to classify participants based on the marital status of their adult children: (1) those with at least one child who is divorced; (2) those with at least one unmarried child aged 30 or older; and (3) a reference group consisting of participants not meeting criteria for the first two categories (i.e., those with adult children who are either married or of an age not deemed suitable for marriage). The group of respondents with both divorced and marriage-delayed children was excluded from the analysis due to its limited representation in the sample (0.57%). Furthermore, we implemented a cumulative stress model, utilizing a metric based on the count of adult children experiencing divorce or delayed marriage.

We defined the exposure variables in this way to reflect our hypothesis that overage singlehood and divorce are significant stressors contributing to parental depression. The third category serves as a baseline group that excludes these stressors. Notably, Categories 1 and 2 focus on the presence of the specified conditions but may also include adult children who are married or not yet of marital age. This approach ensures that the exposure variables align with the theoretical assumptions of the exposure stress model. In addition, we selected 30 as a designated cutoff point for age, aligning with the anticipated age for achieving independence in Chinese Confucian culture, typically marked by milestones such as family establishment. The terms “surplus females” (“Sheng Nv”) and “surplus males” (“Sheng Nan”; Huang, 2014), referring to individuals in China who remain unmarried by the age of 30, have gained widespread usage in public discourse. Existing research has indicated that parents tend to experience heightened stress when their children are in their early 30s and unmarried (Chen & Tong, 2021).

### Outcome

We utilized the 10-item Centre for Epidemiologic Studies—Depression Scale (CES-D) to assess depressive symptoms among the study participants. CES-D-10 examines the frequency of respondents’ experience of depressive symptoms over the past week, employing a Likert scale with categories such as “rarely or none of the time (less than 1 day),” “some or a little of the time (1–2 days),” “occasionally or a moderate amount of time (3–4 days),” and “most or all the time (5–7 days).” Before summing up scores from all items, we reversed Items 5 and 8 scores to align with the rest of the scale. The total CES-D score ranges from 0 to 30, with higher scores indicating more depressive symptoms.

### Confounders

Our analysis incorporated several demographic and lifestyle factors as time-invariant and time-varying confounders. The time-invariant confounders included gender (male, female), age at baseline, number of adult children, educational level, and living area (urban, rural). The educational level was categorized based on the highest qualification attained, with classifications of illiterate, less than elementary school, elementary school, middle school, and high school or above.

Additionally, we considered the following time-varying covariates measured across different waves. The respondents’ marital statuses were dichotomized into two groups: married (including cohabitated) and single (divorced/separated/widowed). Smoking status was categorized as never smoked, ex-smokers, and current smokers. Alcohol drinking was classified as never drinker, occasional drinker, and regular drinker. Annual household expenditure was log-transformed and used as an indicator of economic status. We defined comorbidity based on their self-reported chronic diseases: those without disease, those with one disease, and those with at least two diseases. Several chronic diseases included in this study were hypertension, dyslipidemia, diabetes, cancer, chronic lung disease, liver disease, cardiovascular disease, kidney disease, digestive disease, memory-related disease, rheumatism, and asthma. The respondents were inquired about their adult children’s educational attainments and the variables were dichotomized to indicate whether at least one adult child had completed college.

### Statistical Analyses

In presenting descriptive statistics, categorical variables are expressed as counts with percentages, whereas continuous variables are represented as means with standard deviations. This study employed MSMs to investigate the causal effect of adult children’s marital status on parental depressive symptoms. This model accommodated time-varying confounding.

Assumptions guiding the adoption of MSMs include the following: (1) Exchangeability implies the absence of unmeasured confounding; (2) Positivity ensures a positive probability of exposure to each treatment level; (3) Consistency requires consistency between the observed outcome and the counterfactual outcome under the observed exposure history (Robins et al., 2000). To create the pseudo-population, each participant is assigned a weight based on the probability of their own exposure history (Cole & Hernán, 2008). The censored respondents do not form a representative sample of the overall cohort, leading to selection bias due to informative losses. To mitigate this, we generated censoring weights to account for participants lost to follow-up, reweighting complete cases by the inverse of their probability of remaining in the study (Leyrat et al., 2021). The final weights were calculated by multiplying the stabilized exposure weights and censoring weights. In Model 1, ordinary least squares regression was employed to estimate the association between adult children’s marital status and parental depressive symptoms based on the exposure stress model, adjusting for time-varying and time-invariant confounders in the weighted sample. In the second model, we used the cumulative count of children categorized as divorced or having postponed marriage instead, to measure the cumulative impact of multiple adult children’s marital status. In addition, we explored variations in the association across different groups. Multiplicative terms were included to test the interaction of older adults’ gender, living area, singlehood, and educational level with the exposures in the weighted models. All analyses were conducted using Stata Version 17.

## Results

At the baseline (Year 2011), 13,077 participants took part in the initial survey, and subsequent assessments were conducted with 11,253 participants in Wave 2 (2013), 10,780 in Wave 3 (2015), 9,870 in Wave 4 (2018), and 9,402 in Wave 5 (2020). Table 1 presents the respondents’ baseline characteristics based on their adult children’s marital status. Compared to the parents without divorced and marriage-delayed children, the participants having at least one divorced child or one delaying marriage were more likely to be older (mean age 67 ± 9.4 years), less educated (illiterate and less than elementary education), having more children, divorced/widowed, living in an urban area, reporting having two or more chronic diseases, exhibiting more depressive symptoms. Compared with the reference group, they were less likely to engage in tobacco smoking and alcohol consumption and have lower household expenditures.

**Table 1.** Baseline Characteristics of the Respondents by Their Adult Children’s Marital Status

Variable	Total	Without divorced or marriage-delayed children (*n* = 11,193)	Having marriage-delayed children (*n* = 868)	Having divorced children (*n* = 1,016)	*p*
Gender, *n* (%)					
Female	6,660 (50.9)	5,626 (50.3)	472 (54.4)	562 (55.3)	<.001
Male	6,417 (49.1)	5,567 (49.7)	396 (45.6)	454 (44.7)	
Age (mean, *SD*)	62.33 (8.63)	61.81 (8.50)	63.43 (7.51)	67.08 (9.42)	<.001
Marital status, *n* (%)					
Married/cohabitated	11,197 (85.6)	9,706 (86.7)	715 (82.4)	776 (76.4)	<.001
Separated/divorced/widowed	1,879 (14.4)	1,486 (13.3)	153 (17.6)	240 (23.6)	
Education, *n* (%)					
Illiterate	4,139 (31.7)	3,423 (30.6)	287 (33.1)	429 (42.3)	<.001
Less than elementary school	2,523 (19.3)	2,120 (19.0)	187 (21.5)	216 (21.3)	
Elementary school	2,737 (21.0)	2,355 (21.1)	181 (20.9)	201 (19.8)	
Middle school	2,175 (16.7)	1,923 (17.2)	135 (15.6)	117 (11.5)	
High school and above	1,482 (11.4)	1,352 (12.1)	78 (9.0)	52 (5.1)	
Living area, *n* (%)					
Rural	7,893 (60.4)	6,782 (60.6)	532 (61.3)	579 (57.0)	.429
Urban	5,184 (39.6)	4,411 (39.4)	336 (38.7)	437 (43.0)	
Comorbidities, *n* (%)					
No chronic disease	3,900 (29.8)	3,405 (30.4)	242 (27.9)	253 (24.9)	
One chronic disease	3,897 (29.8)	3,321 (29.7)	261 (30.1)	315 (31.0)	<.001
At least two diseases	5,275 (40.4)	4,464 (39.9)	364 (42.0)	447 (44.0)	
Smoking, *n* (%)					
Never smoke	3,900 (29.8)	6,549 (60.8)	519 (61.9)	605 (61.4)	
Ever smoke	3,897 (29.8)	979 (9.1)	87 (10.4)	121 (12.3)	<.001
Current smoke	5,275 (40.4)	3,245 (30.1)	233 (27.8)	259 (26.3)	
Alcohol drinking, *n* (%)					
Never drink	8,835 (67.6)	7,527 (67.3)	597 (68.9)	711 (70.1)	
Occasionally drink	961 (7.4)	835 (7.5)	57 (6.6)	69 (6.8)	<.001
Regularly drink	3,264 (25.0)	2817 (25.2)	213 (24.6)	234 (23.1)	
Children’s education, *n* (%)					
Not completed college	10,530 (80.7)	8,943 (80.1)	703 (81.0)	884 (87.2)	<.001
At least one completed college	2,516 (19.3)	2,221 (19.9)	165 (19.0)	130 (12.8)	
The number of children (mean; *SD*)	2.93 (1.45)	2.84 (1.42)	3.28 (1.48)	3.58 (1.60)	<.001
Household expenditure (mean; *SD*)	9.53 (1.11)	9.56 (1.10)	9.46 (1.07)	9.31 (1.29)	<.001
CES-D score (mean; *SD*)	8.68 (6.42)	8.41 (6.31)	9.87 (6.60)	10.67 (6.95)	<.001

*Notes*: CES-D = Centre for Epidemiologic Studies—Depression Scale; *SD* = standard deviation. Household expenditure was log-transformed.

Table 2 showed that participants with children who postponed marriage, as well as those with divorced adult children, both exhibited higher levels of depressive symptoms compared to the reference group, which consists of participants with adult children who are either married or of an age not deemed suitable for marriage. The multivariable-adjusted effect estimates (β coefficients, 95% confidence intervals [CIs]) of parental depressive symptoms were 0.83 (0.53, 1.13) for participants with unmarried adult children and 1.55 (1.28, 1.82) for participants with divorced adult children compared to the reference. When adult children’s marital status was included as a continuous variable (Model 2 in Table 2), the coefficient for parental depressive symptoms was 1.10 (95% CI: 0.92–1.28). The associations between grown children’s marital status and parental depressive symptoms were consistent across subgroups according to gender, living areas, educational levels, and singlehood status of the respondents (*P*-value for interaction >.05; Table 3).

**Table 2.** Weighted OLS Regression Estimates of the Effect of Adult Children’s Marital Status on Parental Depressive Symptoms

Variable	Model 1^a^	Model 2^b^
	β (95% CI)	β (95% CI)
Adult children’s marital status		
Without divorced or marriage-delayed children	Ref.	
Having marriage-delayed children	0.83 (0.53, 1.13)	
Having divorced children	1.55 (1.28, 1.82)	
Total number of divorced or marriage-delayed children		1.10 (0.92, 1.28)
Age	−0.05 (−0.06, −0.03)	−0.04 (−0.06, −0.03)
Gender		
Female	Ref.	Ref.
Male	−1.53 (−1.78, −1.27)	−1.55 (−1.81, −1.30)
Education		
Illiterate	Ref.	Ref.
Less than elementary school	0.01 (−0.25, 0.27)	0.04 (−0.23, 0.31)
Elementary school	−0.71 (−0.98, −0.44)	−0.67 (−0.94, −0.39)
Middle school	−1.22 (−1.51, −0.93)	−1.17 (−1.46, −0.87)
High school and above	−2.02 (−2.34, −1.69)	−1.96 (−2.29, −1.64)
Marital status		
Married/cohabitated	Ref.	Ref.
Separated/divorced/widowed	0.91 (0.60, 1.21)	0.84 (0.53, 1.14)
The number of children	0.16 (0.09, 0.24)	0.17 (0.09, 0.24)
Residential area		
Rural	Ref.	Ref.
Urban	−1.34 (−1.53, −1.14)	−1.23 (−1.43, −1.03)
Household expenditure	−0.22 (−0.32, −0.13)	−0.23 (−0.34, −0.13)
Children’s education		
Not completed college	Ref.	Ref.
At least one completed college	−0.89 (−1.11, −0.67)	−0.89 (−1.11, −0.66)
Comorbidities		
No chronic disease	Ref.	Ref.
One chronic disease	1.15 (0.93, 1.37)	1.11 (0.88, 1.34)
At least two diseases	2.60 (2.39, 2.81)	2.72 (2.51, 2.94)
Smoking		
Never smoke	Ref.	Ref.
Ever smoke	0.22 (−0.14, 0.58)	0.20 (−0.15, 0.54)
Current smoke	0.41 (0.17, 0.66)	0.42 (0.18, 0.66)
Alcohol drinking		
Never drink	Ref.	Ref.
Occasionally drink	−0.21 (−0.56, 0.14)	−0.16 (−0.51, 0.20)
Regularly drink	−0.32 (−0.54, −0.09)	−0.25 (−0.48, −0.02)

*Notes*: CI = confidence interval; OLS = ordinary least squares.

^a^Model 1 adjusted for categorical children’s marital status based on the exposure stress model.

^b^Model 2 adjusted for the number of either divorced or marriage-delayed children based on the cumulative stress model.

**Table 3.** Weighted OLS Regression Estimates of the Effect of Adult Children’s Marital Status on Parental Depressive Symptoms, Stratified by Gender, Singlehood, Residence Area, and Education Level

Variable	Adult children’s marital status			*p*_interaction_
	Without divorced or marriage-delayed children β (95% CI)	Having marriage-delayed children β (95% CI)	Having divorced children β (95% CI)	
		
Gender				
Women	Ref.	0.85 (0.44, 1.26)	1.72 (1.36, 2.08)	.34
Men	Ref.	0.78 (0.35, 1.22)	1.30 (0.90, 1.70)	
Parent’s singlehood				
Single	Ref.	0.33 (−0.35, 1.01)	1.34 (0.77, 1.91)	.30
Married	Ref.	0.90 (0.57, 1.22)	1.56 (1.26, 1.85)	
Education				
Illiterate	Ref.	1.05 (0.47, 1.64)	1.93 (1.46, 2.40)	.16
Elementary school	Ref.	0.87 (0.40, 1.33)	1.50 (1.09, 1.91)	
Middle school and above	Ref.	0.65 (0.16, 1.14)	0.99 (0.47, 1.52)	
Living area				
Rural	Ref.	0.96 (0.59, 1.32)	1.75 (1.42, 2.08)	.25
Urban	Ref.	0.63 (0.18, 1.09)	1.21 (0.80, 1.63)	

*Notes*: CI = confidence interval; OLS = ordinary least squares.

## Discussion

Using the longitudinal data in China, we observed that individuals with adult children who experienced divorce exhibited an increase of 1.55 points in CES-D scores, in contrast to parents whose adult children were neither divorced nor had deferred marriage. Furthermore, having adult children who delayed marriage was also linked to higher levels of depressive symptoms in parents. The marital statuses of multiple adult children may have an incremental effect on the mental well-being of their parents, indicating a monotonic exposure–response relationship.

We found that older Chinese individuals whose children are divorced had considerably more depressive symptoms, with the magnitude of this effect estimate being much stronger than the stressor from adult children delaying marriage. Our study in the Chinese context supplements the extensive body of research assessing the impact of children-related challenges on aging parents in Western societies (Birditt et al., 2010; Cichy et al., 2013). A qualitative study based on in-depth interviews with Israeli parents of adult children sheds light on how parents perceive this stage of their role (Levitzki, 2009). Despite the cultural differences, the psychological experiences of parenting adult children align closely with our findings. Parents often view their children as extensions of themselves, and as such, the challenges faced by their children can significantly influence their self-evaluations (Levitzki, 2009).

Our research indicates that the parents with children who delayed marriage (remaining unmarried past the age of 30) showed higher levels of depressive symptoms compared to the reference group. This aligns with the results of a Korean study (Kim et al., 2017); however, it is worth noting that our study did not include parents with a mix of marriage-delayed and divorced children. Our findings contrast a previous study on a subset of parents with children aged 30–44 years (Chen & Tong, 2021). That study reported the association between adult children’s delayed marriage and parental depressive symptoms was not significant, but the son’s overage singlehood increased parental depressive symptoms. The divergence in findings between their study and ours may be attributed to their practice of randomly selecting one child from the household. This approach may overlook the cumulative or interactive effects observed in families that have multiple children who are single or divorced (Knoester, 2003). East Asian countries such as China, Korea, and Japan have long been characterized as distinct from their Western counterparts, highlighting attributes such as extended family co-residence and stronger family ties (Raymo et al., 2015). Such distinctions can be traced back to the shared origin of filial piety in Confucianism, which emphasizes the primary duty of perpetuating the family lineage (Chen & Chen, 2014; Yan, 2011).

Adult children experiencing marital problems or delaying marriage may diverge from their parent’s expectations, giving rise to increased ambivalence for the parents (Pillemer et al., 2007). This conflict was considered to mediate the relationship between the marital status of grown children and parental psychological well-being (Fingerman et al., 2006). The ambivalent reactions in the parent–child relationship could directly compromise the mental health of parents by intensifying emotions of stigma, loss, fear, and concern (Cichy et al., 2013). Deviation from parental expectations might lead parents to perceive, at least in part, that they have failed in their parental roles (Greenfield & Marks, 2006). Although intergenerational stake primarily emphasizes intergenerational differences in the relationship quality, it can also elucidate parents’ vulnerability to their children’s circumstances, given the more significant emotional investment in the parent–child bond in the older generation.

It has been observed that a shift in marriage patterns in post-reform China is characterized by a delayed age of marriage, low marital stability, and high divorce rates (Chen et al., 2021; Yu & Xie, 2015). The expansion of education during economic reforms has improved women’s job prospects and fostered economic independence (Ma & Rizzi, 2017). Additionally, China’s growing embrace of Western values and lifestyles has influenced the likelihood of marriage. Furthermore, urbanization in China, driven by internal migration, has contributed to higher rates of marital dissolution (Chen et al., 2021). The conventional expectation has become less appealing among highly educated young women and has proved increasingly challenging for young men with lower socioeconomic status (Yu & Xie, 2015). In Chinese media, highly educated women of marriageable age who are still unmarried have been derogatorily referred to as “leftover women” (To, 2013), a term that may heighten parental anxiety. On the other hand, a significant gender imbalance with a surplus of males in China leads to a severe marriage squeeze, particularly among males in the lowest social strata (Huang, 2014). Older unmarried sons, often labeled as “forced bachelors,” represent a significant stressor to their parents’ well-being, with reduced financial support potentially acting as a mediating factor (Jin et al., 2015).

Numerous research works addressing the impact of parental well-being have concentrated on only one adult child (Chen & Tong, 2021). Yet, the parent’s mental health may differ if more or even all adult children experience unfavorable marital statuses. Coping with stressors emanating from two children proved to be more challenging than just one child (Ward, 2008). Given that the childbearing period for the majority of participants in the CHARLS study occurred well before the implementation of the one-child policy, resulting in an average family size of approximately three children per family, it is crucial to examine the impact of having multiple divorced or overage single adult children on the mental well-being of aging parents. In addition to the respondents’ demographics, health status, and lifestyle, we also controlled for family size and the educational levels of adult children in the analysis. The likelihood of having at least one child with a marital problem is higher in families with more children, which may influence the effect estimates in the exposure stress model (Fingerman et al., 2012). The educational attainment of grown children dynamically influences marital status and impacts parents’ well-being through intergenerational transfers and associated stigma (Dennison & Lee, 2021).

Evidence suggests that mothers generally anticipate higher levels of support exchange, particularly in the form of expressive support (Sechrist et al., 2014), which includes offering comfort during personal crises or providing advice on adult children’s life decisions, compared to fathers. We hypothesized that parental gender might moderate this effect. Contrary to our expectation, however, our findings revealed that the impact of children’s marital decisions was comparable for both mothers and fathers. This result aligns with prior studies, which have similarly found minimal differences in parental responses to children’s life events based on gender (Fingerman et al., 2008; Knoester, 2003). Several studies have investigated the differential impacts of grown children on mothers and fathers, yielding inconsistent results. Prior research in the United States demonstrated that the marital status of parents may also contribute to variations in susceptibility to stressors from adult children (Greenfield & Marks, 2006). The adult children’s unfavorable marital status may have a stronger effect on single parents because they tend to prioritize their children as their most salient connections (Fingerman et al., 2004). However, our findings indicate no heterogeneity between married and single parents. This could be attributed to the difference in family dynamics between Western societies and China, particularly concerning the role of marriage in the lives of aging parents.

### Strengths and Limitations

We are aware of some potential limitations in this study. First, there is a potential for reporting bias in this study as the information concerning the marital status of adult children relies on reported data from parents rather than directly from the adult children themselves. This issue becomes especially pertinent in cases where adult children might choose to withhold information regarding their marital status from their parents. Second, in our cumulative model, we considered adult children’s delayed marriage and divorce as similar exposures to be counted together. Owing to the limited sample size, it was not feasible to treat these two types of exposures as distinct stressors. However, the results derived from the exposure stress model underscore the importance of considering them separately in future research. Third, the expectation for adult children’s marriage and family values affected by historical circumstances may vary over time. The birth years of respondents in this study vary widely. Still, our study needed more statistical power to fully understand the variations in effects across cohorts despite the evolving family values observed across generations. Fourth, unmarried Chinese women over the age of 30 are under harsher scrutiny and more stigmatized than their male counterparts. However, the data on the gender of adult children are incomplete. Moreover, gender and marital status data are misaligned in some waves, making it difficult to define and analyze “leftover” women and men based on gender-specific age thresholds. Lastly, the variables included in the analysis are considered potential confounding factors suggested by previous literature, but the assumption of “no unmeasured confounding” cannot be empirically verified.

Despite these limitations, it is essential to recognize the strengths of this study. This research entailed a 9-year follow-up period based on large nationally representative data. The innovative use of MSMs allowed for addressing time-dependent confounding and exposures, enabling a causal investigation into the effect of adult children’s marital status on parental mental health.

In summary, this study provides evidence supporting the associations between adult children’s divorce and delayed marriage and parental depressive symptoms in the Chinese context. Furthermore, the marital statuses of multiple adult children may have an incremental effect on the mental well-being of their parents. This research helps uncover culturally significant risk factors for late-life depression, providing insight into the substantial burden of mental health in China. This necessitates the promotion of psychological support and mental health services, alongside the development of targeted preventive strategies, to effectively address the needs of these at-risk older populations.

## Data Availability

The data used in this study were obtained from the China Health and Retirement Longitudinal Study (CHARLS), a publicly available, nationally representative longitudinal survey. Researchers can access CHARLS data through its official website (https://charls.pku.edu.cn/en/) after completing a formal application process. This study was not preregistered.
